# The First Identification in Italy of SARS-CoV-2 Omicron BA.4 Harboring KSF141_del: A Genomic Comparison with Omicron Sub-Variants

**DOI:** 10.3390/biomedicines10081839

**Published:** 2022-07-30

**Authors:** Cinzia Peronace, Rossana Tallerico, Manuela Colosimo, Marco De Fazio, Federica Pasceri, Ilenia Talotta, Giuseppina Panduri, Letizia Pintomalli, Rosaria Oteri, Valeria Calantoni, Maria Teresa Fiorillo, Maria Cristina Caroleo, Rosita Curcio, Vincenza Dolce, Erika Cione, Pasquale Minchella

**Affiliations:** 1 Microbiology and Virology Unit, Pugliese-Ciaccio Hospital, 88100 Catanzaro, Italy; rossana.tallerico@gmail.com (R.T.); manuelacolosimo@hotmail.it (M.C.); marco_2592@yahoo.it (M.D.F.); federicapasceri@gmail.com (F.P.); ilenia.talotta@gmail.com (I.T.); pandurigiuseppina@gmail.com (G.P.); pminchella@aocz.it (P.M.); 2 Unit of Microbiology and Virology, North Health Center ASP 5, 89123 Reggio Calabria, Italy; letizia.pintomalli@libero.it (L.P.); oterirosaria@gmail.com (R.O.); valeriacal@libero.it (V.C.); mariatfiorillo@hotmail.com (M.T.F.); 3 Department of Health Science, University of Catanzaro, 88100 Catanzaro, Italy; mariacristinacaroleo@virgilio.it; 4 Department of Pharmacy, Health, and Nutritional Sciences, University of Calabria, 87036 Rende, Italy; rosita.curcio@unical.it (R.C.); vincenza.dolce@unical.it (V.D.)

**Keywords:** SARS-CoV-2, variant of concern, BA.4, pandemic, KSF141_del, nsp1, spike

## Abstract

The rapid emergence and worldwide detection of the SARS-CoV-2 Omicron variant underscore the importance of robust genomic surveillance systems and prompt information sharing among global public health partners. The Omicron variant has rapidly replaced the Delta variant as a dominating SARS-CoV-2 variant because of natural selection, favoring the variant with higher infectivity and stronger vaccine breakthrough capability. The Omicron variant is also known as B.1.1.529. It has four sub-variants, indicated as BA.1, BA.2, BA.3 and BA.4. Among them, BA.1 is the currently prevailing sub-variant, and BA.2 has been found to be able to alarmingly re-infect patients initially infected by Omicron BA.1. The BA.3 sub-variant is a combination of mutations of BA.1 and BA.2, especially in the spike protein. Today, the BA.4 variant is emerging, which is herein described, and it was the first detected in Italy. Via bioinformatic analysis, we are reporting that the BA.4 that was identified harbors a new mutation, specifically a deletion in the ORF1ab gene, corresponding to KSF141_del in non-structural protein 1 (nsp1), a critical virulence factor able to suppress host translation. The bioinformatics comparison analysis with the other three sub-variants reveals that the deletion was not present before and was never reported until now. Therefore, we can speculate that Omicron BA.4 will become a new dominating “variant of concern” and may also break vaccine protection. Moreover, we show that other proteins are mutated in the BA.4. In particular, seven mutations are recognized in the nucleocapsid (N) protein, and the capability of five different types of rapid antigenic tests are used to identify it.

## 1. Introduction

A new variant of SARS-CoV-2, B.1.1.529 (Omicron) [1,2] was first reported to the World Health Organization (WHO) by South Africa on 24 November 2021 and was designated as a variant of concern [3]. We reported the first case in our region (Calabria, Italy) on 5 December 2021 [4]. The variant carries an unusually high number of mutations, of which 32 are located within the spike (S) protein. The S protein in the key viral component determines the virus’s infectivity and antigenicity. Furthermore, 15 of 32 mutations are located in the spike protein’s receptor-binding region (RBD) that interacts with human cells before cell entry, possibly enhancing the transmissibility [5]. Omicron (B.1.1.529) and four sub-variants of Omicron, which are BA.1 (B.1.1.529.1), BA.2 (B.1.1.529.2), BA.3 (B.1.1.529.3), and BA. 4 (B.1.1.529.4), are closely related variants with a common ancestor [6,7,8]. The data reported in the literature confirm Omicron’s high infectivity [9,10], high vaccine breakthrough rate [11,12] and severe antibody escape rate [13]. Though all the three lineages have spread worldwide, the rate of the spread of these three lineages is different. Of the Omicron sequences submitted to GISAID, the BA.1 lineage is approximately > 98%, the BA.2 is approximately 1% sequence and the BA.3 is around 0.1% sequence (www.gisaid.org accessed on 15 November 2021). Of these three lineages, only BA.1 dominates much more than the other lineages that have ousted Delta. This is likely due to differences in mutations in the spike protein required for virus transmission and host cell entry [14,15]. Liu et al. reported that, of these four lineages, one appears to be the parental lineage of the Omicron (B.1.1.529) variant, and the BA.1 lineage seems to be the closest to this lineage. Instead, BA.2 has significant diversity from the B.1.1.529 and BA.1 lineage, whereas BA.3 has intermediate lineage to BA.1 and BA.2 [16]. No published data are reported in the literature about BA.4 lineage today. In Italy, we first identified by sequencing this lineage on 25 April by depositing it in the ICOGEN Platform by Istituto Superiore di Sanità (ISS). The Omicron lineage BA.4 found in the Calabria Region was isolated from a fully (three doses) vaccinated subject (BioNTech, Pfizer vaccine). Our study aimed to compare this BA.4 sub-variant to the other three sub-variants, together with the capability of five different types of Rapid Antigenic Tests (RATs) to recognize it.

## 2. Materials and Methods

### 2.1. Sample Collection and Viral RNA Extraction

The positive nasopharyngeal and oropharyngeal swab was collected in UTM™ and was extracted for viral nucleic acid purification. A Real-Time (RT) PCR test was carried out with the TaqPath COVID-19 CE-IVD RT-PCR kit (Ref: A48099 Lot: 2101036, Thermo Fisher, Waltham, Massachusetts, USA), which targets the following genes of SARS-CoV-2: (i) open reading frame (ORF)1ab; (ii) nucleocapsid (N) and (iii) spike (S), coupled with QuantStudio 5 DX Thermo-Fisher Real-Time PCR (RT-PCR) (Thermo Fisher, Waltham, MA, USA), as described in our previous study and according to manufacturer [17]. Briefly, a total of 180 µL of the sample was used for RNA extraction by an automated instrument (MGISP-100, MGI, San Jose, CA, USA) using the MGIEasy Nucleic Acid Extraction Kit with superparamagnetic beads technology (MGI, San Jose, CA, USA). Before RNA extraction, 10 μL of Proteinase K was added to each well in a King-Fisher™ Deep 96-well Plate. In addition, 10 μL of the MS2 Phage Control was added to all specimens with 10 μL of magnetic beads. RNA that was extracted by the specimen underwent genomic characterization with the following two methodologies: Sanger-based sequencing by the SeqStudio Genetic Analyzer (Thermo Fisher Scientific, Waltham, MA, USA) and whole-genome based on next-generation sequencing (NGS) by MiSeq System (Illumina, San Diego, CA, USA). The Regional Center performed the latter at the Microbiology and Virology Laboratory of Catanzaro. This workflow was followed in routine practice by accredited laboratories in clinical settings.

### 2.2. Library Preparation, Next-Generation Sequencing and Bioinformatics

RNA that was extracted from the specimen was used for the next library preparation step for next-generation sequencing (NGS) on the Illumina sequencing platform, MiSeq System (Illumina, San Diego, CA, USA). The CleanPlex SARS-CoV-2 FLEX Paragon Genomics Panel performed a reverse transcription of the whole-genome and library preparation (Paragon Genomics, Inc., Hayward, CA, USA). The thermal-cycling or incubation reaction was followed by a library purification using magnetic beads (CleanMag Magnetic Beads). The workflow involved three steps: (i) the first step was cDNA synthesis and purification from purified RNA samples; (ii) the second step was a multiplex PCR reaction that used target-specific primers to targets of interest, thus covering the entire SARS-CoV-2 genome with the 2-pool design, and at this stage, an internal human (host) housekeeping RNA control primer pair was also added; (iii) the third step was a digestion reaction to add sample-level indexes to the generated libraries. The full technote can be found at (https://www.paragongenomics.com/wp-content/uploads/2020/03/UG4001-01_-CleanPlex-SARS-CoV-2-Panel-User-Guide.pdf accessed on 30 April 2022). These libraries were quantified using a Qubit dsDNA HS Assay Kit (Invitrogen by Thermo Fisher Scientific, Waltham, MA, USA). The quality of the library was checked using the DNA high sensitivity assay kit on a Bio-analyser 2100 (Agilent Technologies, Santa Clara, CA, USA), and it was sequenced by a MiSeq platform providing 2 × 250 bp read length data. The SOPHIA DDM Platform analyzed FASTQ reads. The ICOGEN Platform Project obtained clade analyses (at https://irida.iss.it/ accessed on 15 November 2021) integrated with IRIDA (Integrated Rapid Infectious Disease Analysis) and ARIES (Advanced Research Infrastructure for Experimentation in GenomicS) by Istituto Superiore di Sanità (ISS) and the GISAID database. The alignment of FASTQ obtained by NGS data was performed by SnapGene^®^ software (from Insightful Science, San Diego, CA, USA; available at snapgene.com accessed on 20 October 2021). The SNAP gene finder has been developed so far to be easily adaptable to several genomes [18]. The nextstrain GSAID was used with nextstrain/ncov (accessed on 11:46 pm, 25 April 2022) to visualize the current situation worldwide.

### 2.3. Rapid SARS-CoV-2 Antigen Detection Assays

Rapid and accurate tests for SARS-CoV-2 screening are essential to expedite disease prevention and control. Five Rapid Antigenic Tests (RATs) based on a lateral flow immunoassay were carried out according to the manufacturer’s instructions (read at 15 min, except for the one herein listed at #2 that was read at 10 min). These included (1) GeneFinder COVID-19 Ag Plus Rapid Test manufactured by OSANG Healthcare Co., Ltd., Anyang, South Korea; (2) Severe Acute Respiratory Syndrome Coronavirus 2 (SARS-CoV-2) Antigen Detection Kit manufactured by Nanjing Vazyme Medical Technology Co., Ltd., Jiangsu, China; (3) SARS-CoV-2 Antigenic Rapid Test Flowflex manufactured by ACON Biotech (Hangzhou) Co., Ltd., Hangzhou, China; (4) SARS-CoV-2 Antigen manufactured by Lifotronic Technology Co, Ltd., Shenzhen, China; and (5) InstaView COVID-19 Antigen manufactured by SG Medical, Inc., Seoul, South Korea.

## 3. Results and Discussion

The uncertainty of the following SARS-CoV-2 variant will be and remains a significant cause of concern for the World Health Organization (WHO). The viral genome consists of several genes encoding non-structural, structural and accessory proteins, including in the viral genome genes for four structural proteins: the spike surface glycoprotein (S), the envelope (E), the membrane (M) and the nucleocapsid (N), as well as several accessory proteins. For diagnostic and screening monitoring, E, N, S and Open Reading Frame (ORF)1ab genes are the targets most frequently used by the RT–PCR method. ORFs are conserved in all coronavirus genomes and code for structural proteins that create the viral envelope. Omicron is dominant worldwide, and currently, sub-variants are tracked. Here, BA.4 was sequenced by Sanger sequencing as the first level of screening based on the S-gene, which was performed using a standard protocol with 12 commercial primer pairings, described by Paden et al. as first-level genetic screening and full-genome sequencing of SARS-CoV-2 [19]. The data analysis obtained by SeqScape software revealed the Omicron variant. The NGS approach provided 2 × 250 bp read length data. The SOPHIA DDM Platform analyzed FASTQ reads. Then, we described sub-variant information using the Pangolin nomenclatures [20], and the Omicron variant sequences were deposited in the ICOGEN Platform on 25 April 2022 and in GISAID on 26 April 2022. The clade analysis revealed two deletions and 28 mutations on the S-gene. These correspond to the following amino acids on the translated protein: T19I; LPPA24S_del; IHV68I_del; G142D; V213G; S371F; S373P; S375F; T376A; D405N; R408S; K417N; N440K; L452R; T478K; E484A; F486V; Q498R; N501Y; Y505H; D614G; S640F; H655Y; N679K; P681H; N764K; D796Y; Q954H; N969K; and D1146, of which some are in common to other Omicron variants. Interestingly, missense mutation L452R is present only in this sub-variant, as shown in Table 1.

**Figure 1:**
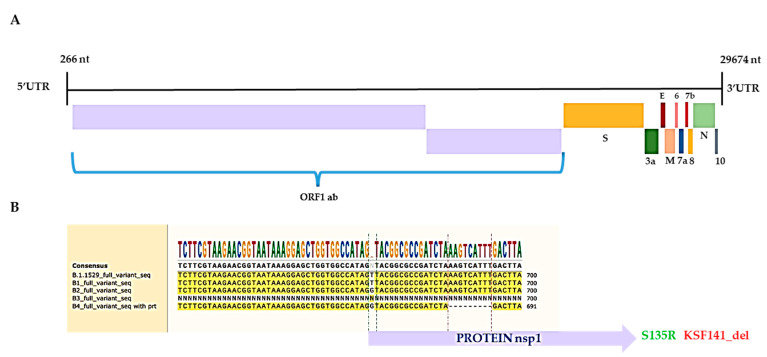
Genome characterization of SARS-CoV-2: (**A**) Schematic representation of SARS-CoV-2 full genome. (**B**) Pictogram of SNAPgene alignment among Omicron lineages.

**Figure 2:**
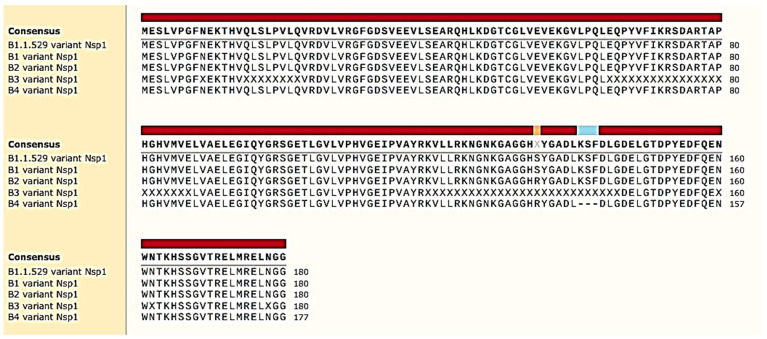
Pictogram of SNAPgene alignment of nsp1 protein portion of ORF1ab protein among Omicron lineages. KSL141_del is in light blue.

As expected, the NGS data pointed out other mutations in additional gene regions of the virus, herein also reported for the ORF1ab protein: S135R; KSF141_del; T24I; F106; G489S; A534; L264F; V290; T327I; L417; T492I; D48; R131; P132H; SGF106_del; E23; I65; S11; P323L; L758; R392C; I42V; T112I; and E145, as shown in Table 1. In addition, the report pointed out in the ORF1ab gene a mutation in S135R, which was already reported in Africa less than 20 days ago (on 4 April 2022 GISAID EPI ISL: EPI_ISL_12243764 for verification), and the KSF141_del deletion instead resulted as a new deletion. The ~30,000 nucleotides (nt) of the virus genome is organized as follows [21]: 5’ to 3’: replicase ORF1ab (~21,000 nt), S, ORF3a (ORF3a protein), E, M, ORF6 (ORF6 protein), ORF7a (ORF7a protein), ORF7b (ORF7b protein), ORF8 (ORF8 protein), N, and ORF10 (ORF10 protein), as is shown in Figure 1A. The important and novel results from our NGS data include evidence of a deletion on the ORF1 ab gene in KSF141_del. ORF1ab is the biggest gene, and in the virus genome, the deletion identified as KSF141_del could be peculiar for this variant, which is classified as BA.4 and which was discovered in Italy. KSF141_del involves nsp1, a critical virulence factor suppressing host translation [21], redirecting the host translation machinery to increase the synthesis of viral proteins, similar to SARS-CoV [22]. The protein nsp1 is usually a highly conserved viral protein for the fundamental biochemical function described above. Furthermore, it directly impairs the host immune response, inducing low antiviral interferons (INFs), types I and III synthesis and release from the cell in animal models and patients [23,24], reflecting the effective viral counteraction of host innate immune responses. It is worth noting that the mutant virus strain harboring an engineered nsp1 with mutations in a critical specific amino acid region (aa 122–130 and aa 155–165) induced more robust antiviral responses and was substantially attenuated in its ability to replicate compared to the wild type virus [25]. The deletion of KSF141_del from the protein was identified here, between these fundamental regions. The KSF141_del nucleic acid deletion was also verified by SNAPgene nucleotide alignment with the other Omicron variant, in which nucleotides in this genetic region are missing, as shown in Figure 1B. KSF141_del was also aligned by SNAPgene protein alignment with the other Omicron variant (Figure 2). Numerous studies have highlighted it as a target for both the development of antivirals and the design of live attenuated vaccines [26].

In the multiple nucleic acid substitutions corresponding, of course, to protein mutations on Omicron BA.4, the presence of seven mutations in the N protein attracted our attention. Compared to other lineages, BA.1, BA.2 and BA.3, which instead harbor four mutations for BA. 1 and BA. 2 and one for BA. 3, this abundance of mutations represents a red flag that could compromise the diagnostic utility of rapid antigen detection tests.

**Figure 3:**
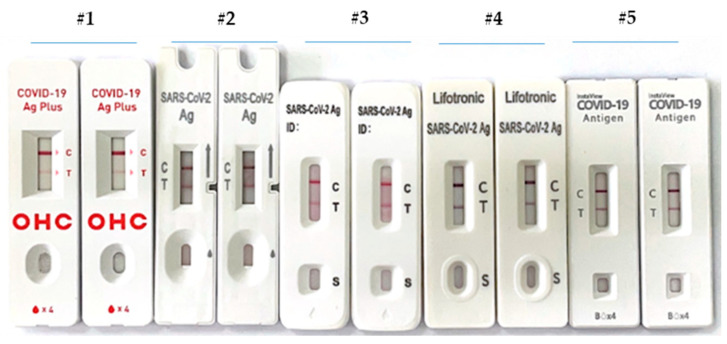
The capabilities of five different test systems, RATs, to recognize Omicron BA.4 from the same sample.

The diagnostic utility of rapid antigen detection diagnostic tests for COVID-19 was recently the object of studies in an elegant systematic review with a meta-analysis by Ghasemi et al. [27]. The main goal of their study was to determine the accuracy of RATs showing that we can use them to identify suspected patients even in the early stage of the disease without concluding if the accuracy of RATs can affect the spread of the COVID-19 virus [28]. Moreover, the authors pushed for more research to determine the efficacy of RATs in detecting the various types of COVID-19 viruses known as variants of concern (VOCs). Therefore, given the emergence of novel SARS-CoV-2 variants of concern, the performance of available diagnostics for these new variants have started to be investigated [29,30]. RATs offer quick, cheap and laboratory-independent results at the point of care. Although their sensitivity is lower compared with RT–PCR, these tests enable consistent detection of high viral loads associated with the presence of infectious viral particles, making them essential public health tools. Therefore, a small aliquot of the collected specimen was processed through a RAT device based on a lateral flow immuno-chromatographic assay. We tested five RATs, reporting their capability to recognize new BA.4 variants, harboring the following mutations on the nucleocapsid (N) protein: P13L; GERS30G_del; P151S; R203K; G204R; S413R; and S416L. The capabilities of the lateral flow immunoassay are presented in Figure 3 and are shown as follows: (1) GeneFinder COVID-19 Ag Plus Rapid Test manufactured by OSANG Healthcare Co., Ltd., Anyang, South Korea; (2) Severe Acute Respiratory Syndrome Coronavirus 2 (SARS-CoV-2) Antigen Detection Kit manufactured by Nanjing Vazyme Medical Technology Co., Ltd., Jiangsu, China; (3) SARS-CoV-2 Antigenic Rapid Test Flowflex manufactured by ACON Biotech (Hangzhou) Co., Ltd., Hangzhou, China; (4) SARS-CoV-2 Antigen manufactured by Lifotronic Technology Co, Ltd., Shenzhen, China; and (5) InstaView COVID-19 Antigen manufactured by SG Medical, Inc., Seoul, South Korea.

The N protein from SARS-CoV-2 is recognized by capturing antigen-conjugate gold particle complexes. They migrate across a reaction area coated by antibodies to nucleocapsid proteins. The positive results display two colors related to control (C) and test (T) lines, whereas only one line in the C area is present for the negative ones. The colored test (T) line’s intensity depends on the amount of SARS-CoV-2 N antigen present in the sample. The devices showed positive results, indicating how RATs with high sensitivity and specificity can represent an excellent screening method for any Omicron variants, including BA.4. This is important, especially in high-prevalence areas of infection [31,32]. Our results show that device #1 looks less sensitive to other RAT tests. Device #4 looks the best. However, as with all variants, a lag exists between infection and more severe outcomes, and symptoms are expected to be milder in vaccinated persons and in those with previous SARS-CoV-2 infections than in unvaccinated persons [20,32]. Although the vaccine produces a whole array of antibodies against the RBD-S spike protein, many unknown mutations are associated with the Omicron variant; therefore, partial immune escape may be expected [33,34]. In this view, the BA.4 sub-variant identified here, harboring the KSF141_del, could be the light at the end of the dark. More studies are needed to better understand Omicron transmissibility, immune escape potential, disease severity and the role of other available diagnostic and therapeutic countermeasures.

## 4. Conclusions

The clinical presentation of COVID-19 is non-specific. To limit the spread of the SARS-CoV-2 virus, an accurate diagnosis with a robust method is needed, even in light of reinfection due to the viral genome’s continuous evolution that leads to protein mutation. We can speculate that Omicron BA.4 will become a new dominating variant of concern and may also break vaccine protection. The recent deletion in the ORF1ab gene linked to the nsp1 protein indicates an exciting target for developing live attenuated vaccines, since it is in a critical region of nsp1. The limit of our discovery regards the impossibility of growing the viral strain with the deletion that is pointed out here, due to concerning biosafety levels in our laboratory (we are in class 2). Therefore, we urge the scientific community, having at least a biosafety level class 3, to perform in vitro tests on this sub-strain. Then, in our opinion, we may use it to produce a live attenuated vaccine. Moreover, we have shown that other proteins are mutated in BA.4. In particular, seven mutations are recognized in the nucleocapsid (N) protein, and the capabilities of five different types of rapid antigenic tests can be used to identify it.

## Figures and Tables

**Table 1:** Amino acid mutations in the genome of Omicron sub-lineages were obtained by whole-genome sequencing.

Protein Mutations								
	ORF1ab	S	ORF3a	E	M	ORF6	ORF7b	N
**BA.1.1.529**	A555; K38R; F106; SL1265I_del; T492I; P132H; LSGF105F_del; I189V; V57; P323L; N600;	A67V; IHV68I_del; T95I; GVYY142D_del; NL211I_del; D215EPED; R346K; S371L; S373P; S375F; K417N; N440K; G446S; F456; T547K; D614G; H655Y; N679K; P681H; N764K; N856K; Q954H; N969K; D1146	T64		D3G; Q19E; A63T;		L18	P13L; GERS30G_del; R203K; G204R;
**BA.1**	E563D; K38R; F106; SL1265I_del; T492I; P132H; LSGF105F_del; I189V; V57; P323L; N600	A67V; IHV68I_del; T95I; GVYY142D_del; NL211I_del; D215EPED; S371L; S373P; S375F; K417N; N440K; G446S; T547	T64	T9I	D3G; Q19E; A63T	R20	L18	P13L; GERS30G_del; R203K; G204R
**BA.2**	S135R; T24I; F106; G489S; A534; A1526V; L264F; V290; T327I; T492I; D48; R131; P132H; SGF106_del; F251L; R252T; Y253S; I65; S11; P323L; L758; I258; R392C; I42V; T112I; E145	T19I; LPPA24S_del; G142D; V213G; D405N; R408S; K417N; N440K; S477N; T478K; E484A; Q493R; Q498R; N501Y; Y505H; T547K; D614G; H655Y; N679K; P681H; N764K; D796Y; Q954H; N969K; D1146	T64; T223I	T9I	Q19E; A63T; F112	R20; D61L	L18	P13L; GERS30G_del; R203K; G204R
**BA.3**	R27C; K38R; F106; L264F; V290; T327I; R131; P132H; SGF106_del; P323L; L758; R392C; I42V; E145	T19I; LPPA24S_del; G142D; S371F; S373P; S375F; T376A; N440K; F456; D614G; H655Y; N679K; P681H; D796Y; Q954H	T64	T9I	F112	D61L	L18	P13L
**BA.4**	S135R; **KSF141_del**; T24I; F106; G489S; A534; L264F; V290; T327I; L417; T492I; D48; R131; P132H; SGF106_del; E23; I65; S11; P323L; L758; R392C; I42V; T112I; E145	T19I; LPPA24S_del; IHV68I_del; G142D; V213G; S371F; S373P; S375F; T376A; D405N; R408S; K417N; N440K; **L452R**; T478K; E484A; F486V; Q498R; N501Y; Y505H; D614G; S640F; H655Y; N679K; P681H; N764K; D796Y; Q954H; N969K; D1146	T64; T223I	T9I	Q19E; A63T; F112	R20; D61L	**L11F**; L18	P13L; GERS30G_del; **P151S**; R203K; G204R; **S413R**; **S416L**

## Data Availability

Data is contained within the article recorded at the Department of Microbiology and Virology, Pugliese Ciaccio’s Hospital, Catanzaro, Italy, and at the Unit of Micro-biology and Virology, North Health Center ASP 5, Reggio Calabria, Italy.
